# Modelling the impact of contact tracing of symptomatic individuals on the COVID-19 epidemic

**DOI:** 10.6061/clinics/2021/e2639

**Published:** 2021-03-19

**Authors:** Marcos Amaku, Dimas Tadeu Covas, Francisco Antonio Bezerra Coutinho, Raymundo Soares Azevedo, Eduardo Massad

**Affiliations:** I Laboratorio de Investigacao Medica (LIM01), Faculdade de Medicina FMUSP, Universidade de Sao Paulo, Sao Paulo, SP, BR.; II Faculdade de Medicina Veterinaria e Zootecnia, Universidade de Sao Paulo, Sao Paulo, SP, BR.; III Instituto Butantan, Sao Paulo, SP, BR.; IV Escola de Matematica Aplicada, Fundacao Getulio Vargas, Rio de Janeiro, RJ, BR.

**Keywords:** COVID-19, SARS-CoV-2, Contact Tracing, Mathematical Model

## Abstract

**OBJECTIVES:**

With the declining numbers of coronavirus disease 2019 (COVID-19) cases in the state of São Paulo, Brazil, social distancing measures have gradually been lifted. However, the risk of a surge in the number of cases cannot be overlooked. Even with the adoption of nonpharmaceutical interventions, such as restrictions on mass gatherings, wearing of masks, and complete or partial closure of schools, other public health measures may help control the epidemic. We aimed to evaluate the impact of the contact tracing of symptomatic individuals on the COVID-19 epidemic regardless of the use of diagnostic testing.

**METHODS:**

We developed a mathematical model that includes isolation of symptomatic individuals and tracing of contacts to assess the effects of the contact tracing of symptomatic individuals on the COVID-19 epidemic in the state of São Paulo.

**RESULTS:**

For a selection efficacy (proportion of isolated contacts who are infected) of 80%, cases and deaths may be reduced by 80% after 60 days when 5000 symptomatic individuals are isolated per day, each of them together with 10 contacts. On the other hand, for a selection efficacy of 20%, the number of cases and deaths may be reduced by approximately 40% and 50%, respectively, compared with the scenario in which no contact-tracing strategy is implemented.

**CONCLUSION:**

Contact tracing of symptomatic individuals may potentially be an alternative strategy when the number of diagnostic tests available is not sufficient for massive testing.

## INTRODUCTION

The first case of coronavirus disease 2019 (COVID-19) in Brazil was reported on February 26, 2020, in the state of São Paulo, the most populous Brazilian state with 44,639,899 inhabitants (1). Genome sequencing and phylogenetic analyses corroborate multiple importations of the virus from Italy, followed by local spread (2). Since October 30, 2020, 1,113,788 cases and 39,255 deaths were reported in Brazil (3), the largest numbers in Latin America (4).

Isolation, quarantine, social distancing, and community containment are important, nonpharmaceutical public health interventions to control the explosive escalation of COVID-19 (5). Liberal testing, followed by contact tracing and isolation of all persons who tested positive, has direct and clear benefits (6).

The World Health Organization recommends a combination of rapid diagnosis, immediate isolation of cases, rigorous tracking, and precautionary self-isolation of close contacts (6). In a previous paper, we analyzed the impact and costs of test-trace-quarantine strategies (7). Here, we set out to model the effects of a contact-tracing strategy of symptomatic individuals on controlling the spread of COVID-19 regardless of the use of diagnostic testing. This may be an alternative strategy for regions with limited availability of diagnostic tests.

## METHODS

### The model

The model is based on a modified version of the susceptible-exposed-infectious-recovered model (7,8) and considers that the population at time *t* is divided into the following several categories: susceptible individuals, *S*(*t*); isolated susceptible individuals, *Q_s_*(*t*); susceptible individuals previously isolated, *S_T_*(*t*); exposed individuals, *E*(*t*); asymptomatic/oligosymptomatic individuals, *A*(*t*); symptomatic individuals, *I*(*t*); isolated infected individuals, *Q*(*t*); hospitalized individuals, *H*(*t*); individuals with severe disease hospitalized in intensive care units (ICUs), *G*(*t*); and recovered individuals, *R*(*t*).

**Figure 1:**
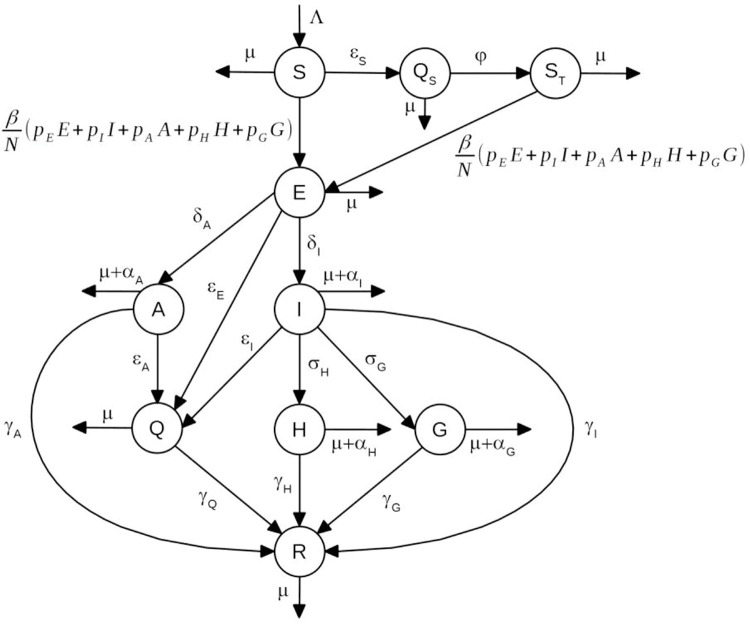
Schematic representation of the model compartments.

A schematic representation of the model is shown in Figure 1.

The dynamics of individuals between compartments may be described as follows:

- Susceptible individuals, *S*(*t*), grow with a birth rate Λ(*t*) and may either acquire the infection with contact rate *β* or be isolated at constant rate *ε_S_* (*i.e.*, *ε_S_* individuals isolated per unit time).
- Isolated susceptible individuals, *Q_S_*(*t*), after a period of l/*φ*, are moved to compartment *S_T_*(*t*).
- Once infected, susceptible individuals, that is, *S*(*t*) and *S_T_*(*t*), move to the state of exposed individuals, denoted by *E*(*t*).
- Exposed individuals may evolve into symptomatic individuals, *I*(*t*), with rate *δ_I_*, or evolve into asymptomatic/oligosymptomatic individuals, denoted by *A*(*t*), with rate *δ_A_*, and may be isolated at constant rate *ε_E_*.
- Infectious individuals, *I*(*t*), may evolve into one of two states: hospitalized individuals denoted by *H*(*t*), with rate *σ_H_*, or into a state in which individuals develop severe disease and are admitted to ICUs, denoted by *G*(*t*), with rate *σ_G_*. Infectious individuals, *I*(*t*), may be isolated at constant rate *ε_I_* and may also die of the disease, with rate *α_I_*.
- Asymptomatic individuals, *A*(*t*), may be isolated at constant rate *ε_A_*.
- Individuals in the states *A*(*t*), *H*(*t*), and *G*(*t*) may die of the disease, with rates *α_A_*,*α_H_*, and *α_G_*, respectively.
- All individuals who acquired the infection and did not die of the disease recover to a new state, denoted by *R*(*t*), with rates *γ_I_*,*γ_A_*,*γ_H_*, and *γ_G_*, as depicted in Figure 1.
- Isolated infected individuals are moved to a state denoted by *Q*(*t*). Since these individuals are isolated from the rest of the population, they do not transmit the virus and will eventually recover from the infection, with rate *γ_Q_*.
- All individuals may die from natural causes, with rate *μ*.
- We assumed that the population birth rate, Λ(*t*), was equal to the natural mortality of the population, disregarding disease-induced mortality.
- The fractions *p_E_*, *p_I_*, *p_A_*, *p_H_*, and *p_G_* of exposed, symptomatic, asymptomatic, hospitalized, and severe (ICU patients) individuals, respectively, can transmit the infection.

The following set of differential equations describes the model dynamics.



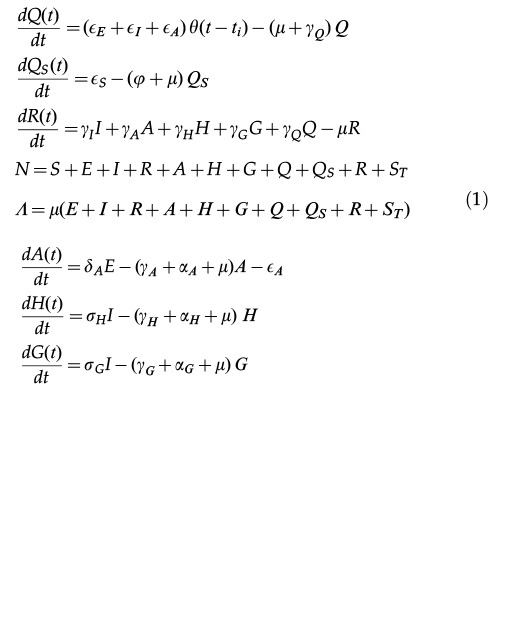



The basic reproduction number of system (1) is given by,



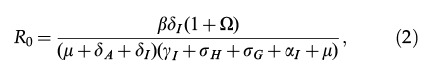



where



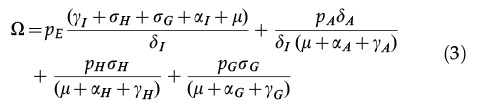



The incidence of infection is given by



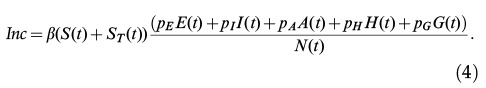



The total number of reported cases is obtained by multiplying the number of infected individuals by a notification ratio *K*(*t*).



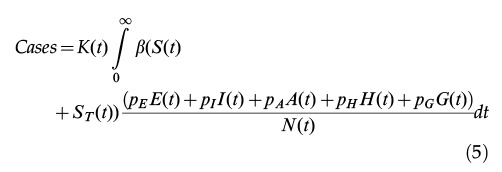



The total number of COVID-19-related deaths is given by



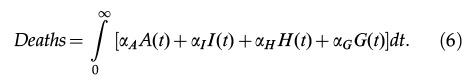



Finally, the total number of isolated individuals is given by



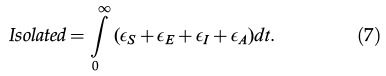



If the number of symptomatic individuals in a certain time interval, Δ*t*, is less than *ε_I_*Δ*t*, only the available symptomatic individuals are isolated together with their contacts. A similar procedure is adopted for the number of susceptible and asymptomatic individuals in the compartments *S*, *E*, or *I* when they are below *ε_S_*Δ*t*, *ε_E_*Δ*t*, or *ε_A_*Δ*t*, respectively.

### Fitting procedure

We used the fitting procedure proposed by Amaku et al. (7) and described it as follows.

Data on the cumulative numbers of reported cases and deaths were obtained from Fundação Sistema Estadual de Análise de Dados do Estado de São Paulo (Seade). Data on the number of ICU patients were obtained from Sistema de Monitoramento Inteligente do Estado de São Paulo. A fitting procedure based on the Levenberg-Marquardt non-linear least-squares algorithm was used to fit the model’s parameters simultaneously to the data on cases, deaths, and ICU patients. We used the R package minpack.lm (9).

We assumed that the potentially infective contact rate, notification ratio, and ICU admission rate change every 10 days.

The parameter values used are shown in Table 1.

Model projections for future dates were obtained by keeping the fitted values of the parameters fixed from the last date observed in the data.

### Contact-tracing (CT) strategy

A number, *ε_I_*, of symptomatic individuals are isolated per unit time. The number, *c*, of contacts of each symptomatic individual are also isolated. We varied both *ε_I_* and *c*. Isolated individuals remain in isolation for 14 days.

Assuming that a fraction of the isolated contacts may be susceptible or recovered, we defined selection efficacy as the proportion of isolated contacts who are infected (asymptomatic or symptomatic individuals).

We calculated the efficacy of the CT strategy by subtracting from 1 the result of the division of the cumulative number of cases by the number of cases in the baseline scenario, in which CT is not performed.

We assumed an initial condition with 15%, 83%, and 2% of recovered, susceptible, and infected individuals, respectively. These estimates are consistent with the model projections for the beginning of August 2020 in the state of São Paulo. Among the infected individuals, an asymptomatic-to-symptomatic ratio of 5 and a ratio of asymptomatic-to-exposed (from compartments A and E) of 9 were assumed.

### Sensitivity analysis

A sensitivity analysis was conducted using a Monte Carlo method to sample parameter values and a partial rank correlation coefficient (PRCC) estimation to measure the strength of association between an input parameter and an output variable after the linear effects on the output variable of the remaining inputs were discounted (10,11). Parameter values were sampled using a Monte Carlo sampling method, assuming a uniform distribution for each parameter. The following input parameters were included in the analysis: proportions of susceptible (*fS*), infected (*fI*), and recovered (*fR*) individuals in the initial condition; number of isolated symptomatic individuals (*ε_I_*) per unit time; number of contacts (*c*) of each symptomatic individual isolated; the selection efficacy (*eff*); and the asymptomatic-to-symptomatic ratio (*r_AS_*). The ranges of parameter values used in the sensitivity analysis are shown in Table 2. The cumulative number of cases after 60 days was used as the output variable.

## RESULTS

**Figure 2:**
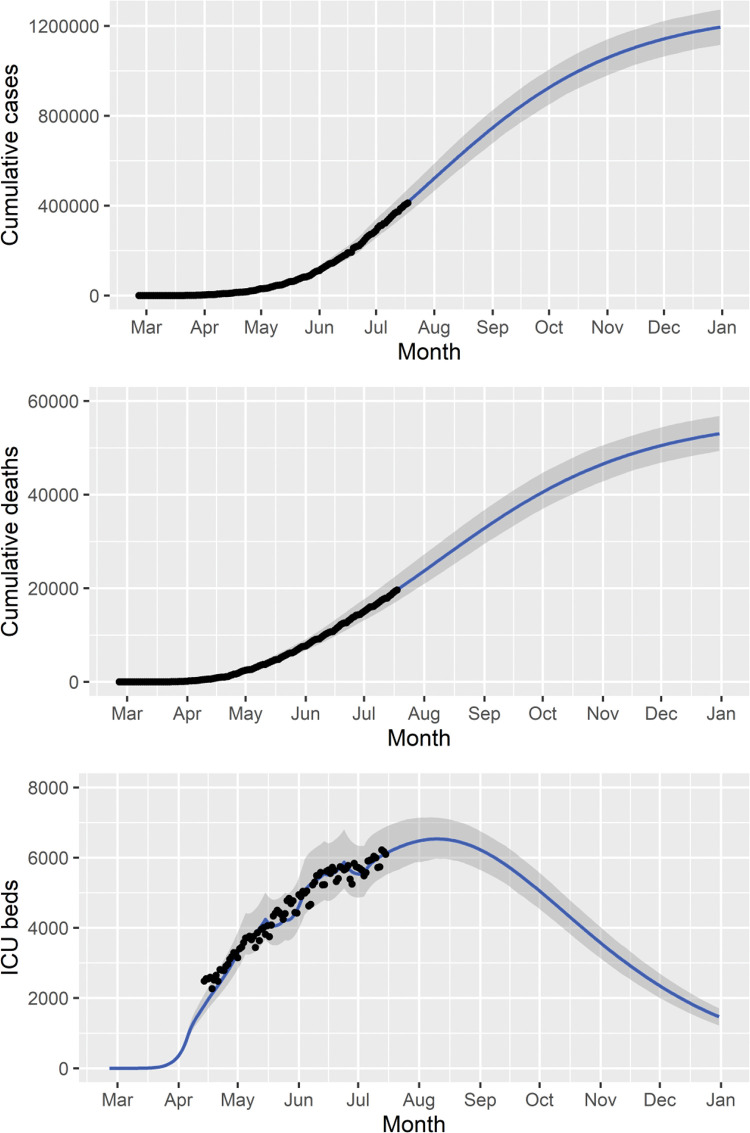
Cumulative numbers of reported cases and deaths, number of ICU patients (black dots), and the corresponding fitted model (blue lines). The solid lines and shaded area correspond to median values and 95% probability intervals, respectively.

We fitted the model parameters simultaneously to the data on the cumulative number of reported cases, deaths, and ICU patients (Figure 2) in the state of São Paulo until July 18, 2020. To estimate a 95% probability interval (shaded area in Figure 2), we assumed a normal distribution for the contact rate with a standard deviation of 1.0%.

**Figure 3:**
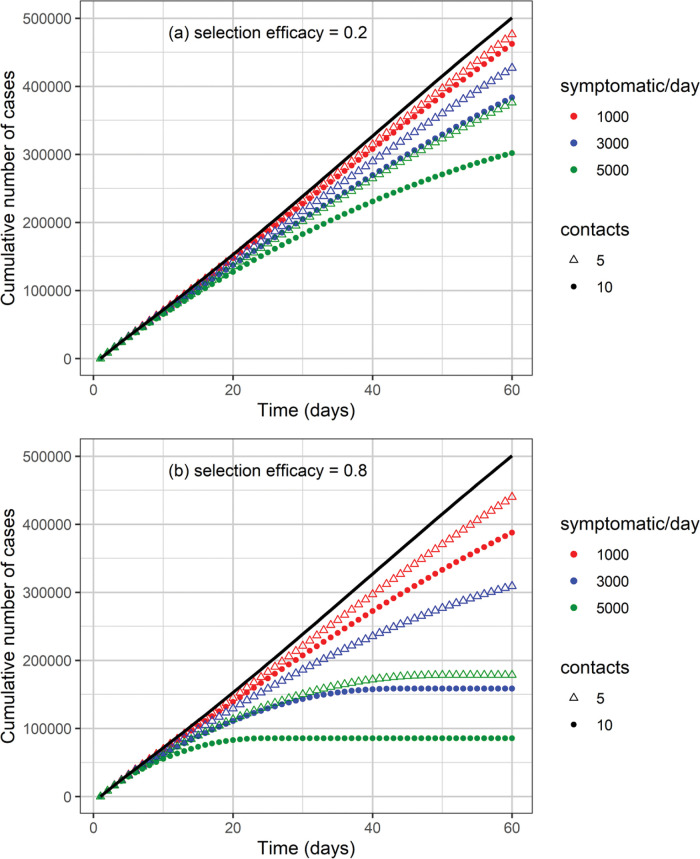
Cumulative number of cases as a function of time for different numbers of isolated symptomatic individuals per day, isolated contacts, and selection efficacies of (a) 20% and (b) 80%. The solid black line shows the effect that would be observed if no isolation strategy is used.

**Figure 4:**
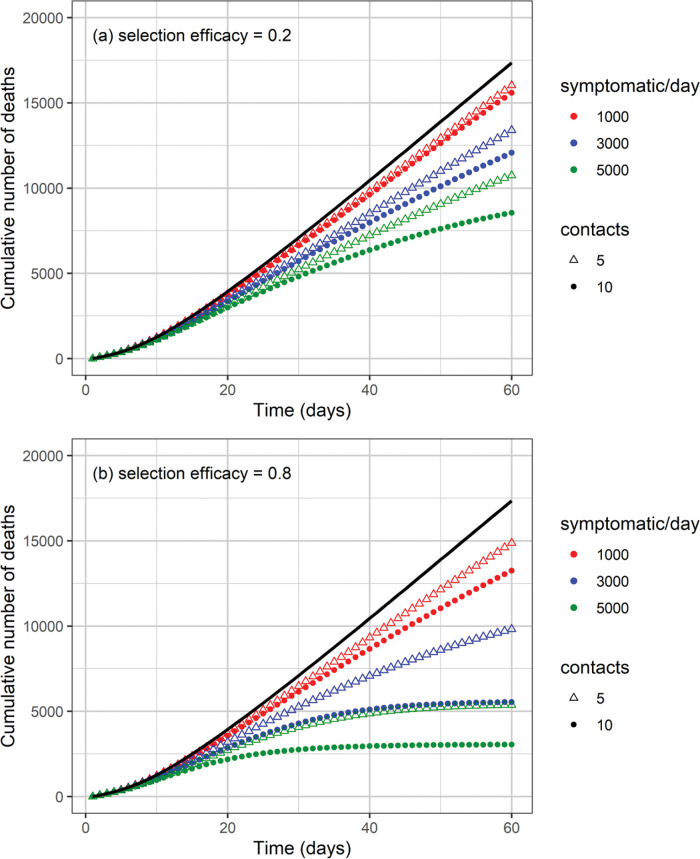
Cumulative number of deaths as a function of time for different numbers of isolated symptomatic individuals per day, isolated contacts, and selection efficacies of (a) 20% and (b) 80%. The solid black line shows the effect that would be observed if no isolation strategy is used.

**Figure 5:**
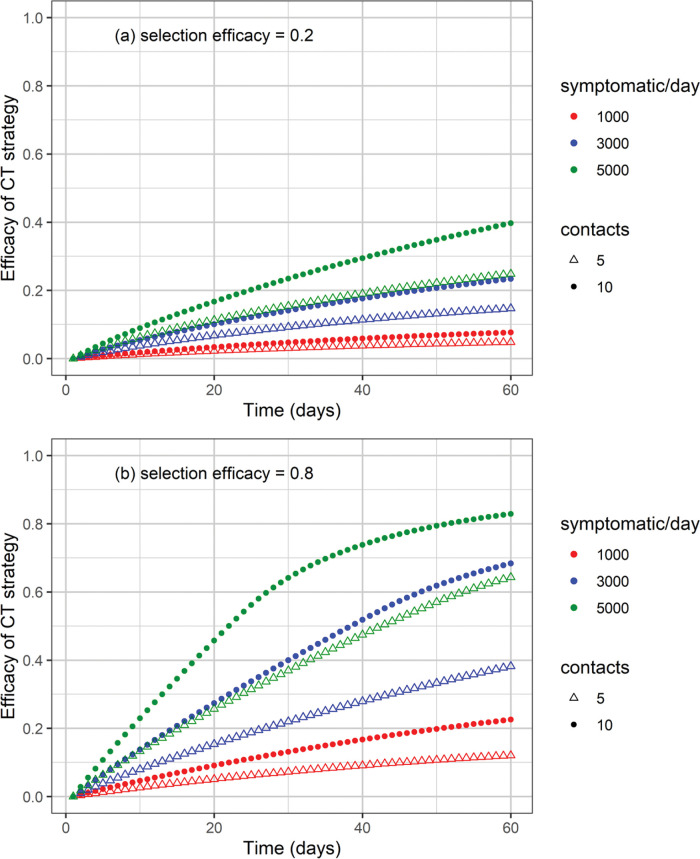
Efficacy of the CT strategy, defined as 1 minus the ratio of the number of cases under a CT strategy divided by the number of cases without CT strategy, as a function of time for different combinations of symptomatic individuals isolated per day, number of isolated contacts, and selection efficacies of (a) 20% and (b) 80%.

**Figure 6:**
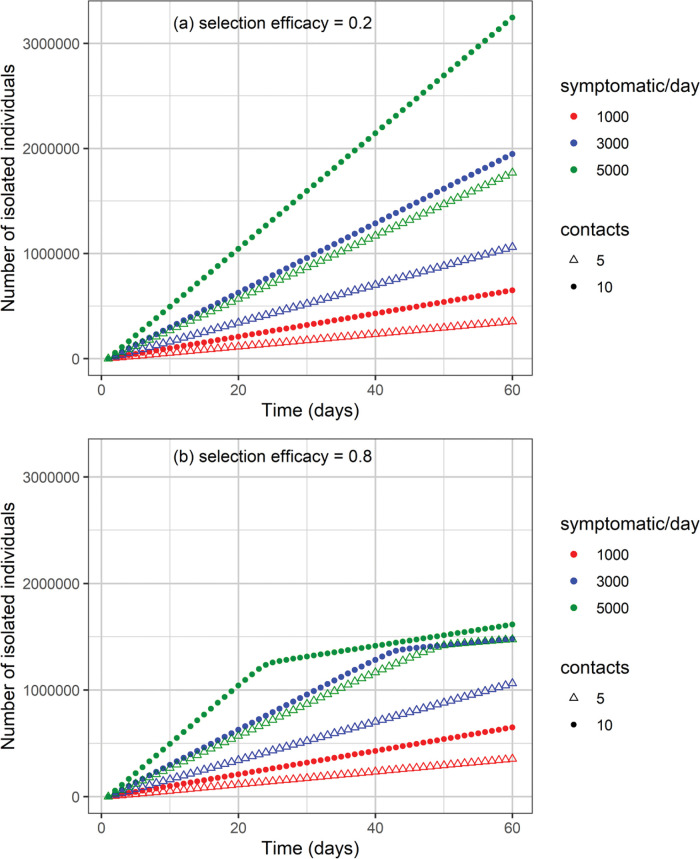
Cumulative number of isolated individuals as a function of time for different combinations of isolated symptomatic individuals per day, isolated contacts, and selection efficacies of (a) 20% and (b) 80%.

The cumulative number of cases and deaths over time for different numbers (*i.e.,* 1000, 3000, and 5000) of symptomatic individuals isolated per day and their contacts (5 or 10 contacts per symptomatic individual) is shown in Figures 3 and 4, respectively. Selection efficacies of 20% and 80% were also considered. The solid line shows the results when no CT strategy was used. The efficacy of the CT strategies and the number of isolated individuals are shown in Figures 5 and 6, respectively.

The higher the number of symptomatic individuals isolated per day, the lower the cumulative number of cases and deaths (Figures 3 and 4). For instance, when 5000 symptomatic individuals are isolated per day (each of them together with 10 contacts), the number of cases and deaths are reduced by approximately 40% and 50%, respectively. This can be compared with the scenario in which the CT strategy is not implemented, for a selection efficacy of 20% and period of 60 days from the initiation of the CT strategy. For a selection efficacy of 80%, the number of cases and deaths is reduced by approximately 80%.

As the calculation of the efficacy of the CT strategy is based on a reduction in the number of cases, for the scenarios described in the previous example, the efficacy of the CT strategy is 40% for a selection efficacy of 20% (Figure 5). For a selection efficacy of 80%, the efficacy of the CT strategy is approximately 82%.

When the selection efficacy is low (20%), the number of isolated individuals may be as high as 3.2 million after 60 days for the strategy involving 5000 symptomatic individuals isolated per day together with 10 contacts for each individual (Figure 6). On the other hand, when the selection efficacy is high (80%), approximately 1.6 million individuals are isolated after 60 days.

**Figure 7:**
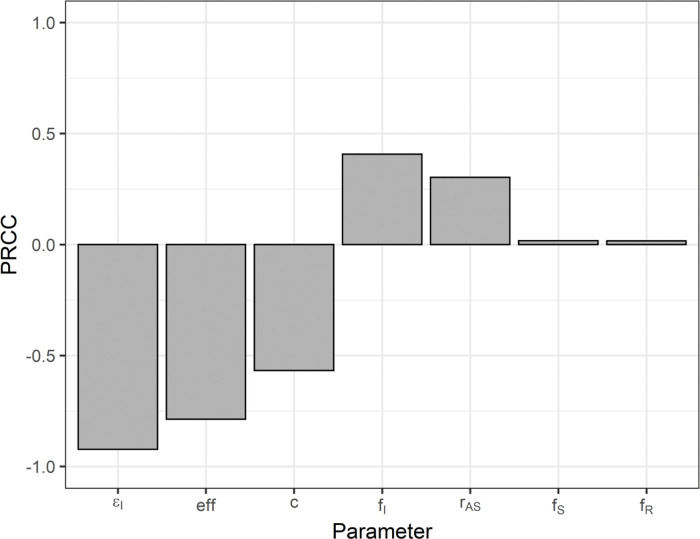
Partial rank correlation coefficients for the number of cumulative cases as the output variable and the following input variables: number of isolated symptomatic individuals (*ε_I_*); selection efficacy (*eff*); number of contacts (*c*); initial proportion of infected (*f_I_*), susceptible (*f_S_*), and recovered (*f_R_*) individuals; and asymptomatic-to-symptomatic ratio (*r_AS_*).

The PRCC values are shown in Figure 7. The sign of the PRCC is related to the qualitative relationship between the input parameter and the output variable (number of cumulative cases). The number of cumulative cases decreases as the number of isolated symptomatic individuals (*ε_I_*), selection efficacy (*eff*), and number of contacts (*c*) increase; thus, the PRCC values are negative. The positive PRCC values for the initial proportion of infected individuals (*f_I_*) and the asymptomatic-to-symptomatic ratio (*r_AS_*) imply that, when these parameters increase, the number of cumulative cases also increases. The PRCC values for the initial proportion of susceptible (*f_S_*) and recovered (*f_R_*) individuals are positive, but closer to zero (low correlation) when these two initial-condition values are in the ranges shown in Table 2.

## DISCUSSION

We modelled the impact of a strategy based on contact tracing of symptomatic individuals on the COVID-19 epidemic in the state of São Paulo, Brazil. This strategy has lower costs when compared to a test-trace-and-quarantine strategy (7). It may potentially be an alternative strategy when the number of diagnostic tests available is not sufficient for massive testing.

In the sensitivity analysis, we observed that the reduction in the number of cumulative cases was more sensitive to the number of isolated symptomatic individuals, selection efficacy, and number of contacts, in decreasing order of the PRCC. An increase in the number of isolated symptomatic individuals and their contacts poses logistical challenges and associated costs. These costs, however, are likely to be lower than those of a test-trace-and-quarantine strategy (7).

The higher the selection efficacy, the higher the efficacy of the CT strategy (Figure 5). The use of high-performance diagnostic tests would likely increase the selection efficacy. However, without the use of diagnostic tests, one could think that tracing of close contacts of symptomatic individuals, such as household members or coworkers, would probably increase the selection efficacy, thus increasing the overall efficacy of the CT strategy.

Optimizing tracing coverage and minimizing tracing delays, for example, with app-based technology, further enhance contact-tracing effectiveness, as pointed out by Kretzschmar et al. (12). As discussed by Bilinski et al. (13), the benefits of contact tracing depend on adherence to isolation and quarantine by individuals who are traced. The adherence may be enhanced by measures such as out-of-home accommodation, income replacement, and social support (13).

A limitation of this analysis is that we assumed that the isolated symptomatic individuals are infected by the severe acute respiratory syndrome coronavirus 2 (SARS-CoV-2) and not by any other virus that could cause similar symptoms. However, this limitation would be less important in a scenario in which a substantial proportion of respiratory infections is being caused by SARS-CoV-2. Nevertheless, one could interpret the number of isolated symptomatic individuals as an effective number of individuals infected by SARS-CoV-2 who should be isolated to observe the outcomes of the model.

## CONCLUSION

We evaluated the impact of contact tracing of symptomatic individuals and their contacts on the number of cases and deaths related to COVID-19. Depending on the number of symptomatic individuals isolated per day and the efficacy of selecting infected (asymptomatic) contacts for isolation, the overall efficacy of the contact-tracing strategy can be high. For instance, for a selection efficacy of 80%, the numbers of cases and deaths may be reduced by 80% after 60 days when 5000 symptomatic individuals are isolated per day, each of them together with 10 contacts. On the other hand, for a selection efficacy of 20%, the numbers of cases and deaths may be reduced by approximately 40% and 50%, respectively, compared with the scenario in which no contact-tracing strategy is implemented. Thus, contact tracing of symptomatic individuals may potentially be an alternative strategy when the number of diagnostic tests available is not sufficient for massive testing.

## AUTHOR CONTRIBUTIONS

Amaku M, Covas DT, Coutinho FAB, Azevedo RS and Massad E participated in the modelling design, discussion of the subject, and writing and revision of the manuscript.

## Figures and Tables

**Table 1:** Parameters used in the model.

Parameter	Description	Value
*β*(*t*)	Potentially infective contact rate	Fitted (changes over time)
*p_E_*	Infectivity of exposed individuals	0.4*
*p_I_*	Infectivity of symptomatic individuals	1.0*
*p_A_*	Infectivity of asymptomatic individuals	1/3*
*p_H_*	Infectivity of hospitalized individuals	0.01*
*p_G_*	Infectivity of ICU patients	0.01*
*μ*	Natural mortality rate (life expectancy of 70 years)	3.91×10^-5^ days^-1^ *
*δ_I_*	Rate of evolution from exposed to infected	1/2 day^-1^ *
*δ_A_*	Rate of evolution from exposed to asymptomatic	1.45 day^-1^ **
*γ_I_*	Rate of recovery from infected	1/3 day^-1^ *
*γ_A_*	Rate of recovery from asymptomatic	1/14 day^-1^ *
*γ_H_*	Rate of recovery from hospitalized	1/10 day^-1^ *
*γ_G_*	Rate of recovery from ICU	0.06752 day^-1^ **
*γ_Q_*	Rate of recovery from isolated	1/14 day^-1^ *
*α_I_*	Disease-induced mortality rate for infected individuals	5×10^-4^ day^-1^ *
*α_A_*	Disease-induced mortality rate for asymptomatic individuals	0*
*α_H_*	Disease-induced mortality rate for hospitalized individuals	2.2012×10^-4^ day^-1^ **
*α_G_*	Disease-induced mortality rate for ICU patients	Fitted (changes over time)
*ε_S_*	Isolation rate of susceptible individuals	Variable
*ε_E_*	Isolation rate of exposed individuals	Variable
*ε_I_*	Isolation rate of symptomatic individuals	Variable
*ε_A_*	Isolation rate of asymptomatic individuals	Variable
*σ_H_*	Hospitalization rate	1.973×10^-2^ day^-1^ **
*σ_G_*	ICU admission rate	Fitted (changes over time)
*φ*	Rate of change from compartment *Q_S_*(*t*) to *S_T_*(*t*)	1/14 day^-1^ *
*K*(*t*)	Notification ratio	Fitted (changes over time)
Λ(*t*)	Birth rate	Changes over time

* assumed;

** fitted.

**Table 2:** Ranges of parameter values used in the sensitivity analysis. The output variable is the cumulative number of cases after 60 days, and the input parameters are described in the table. Parameter values were sampled using a Monte Carlo sampling method assuming a uniform distribution.

Input parameter	Description	Range
*f_S_*	Proportion of susceptible individuals in the initial condition	Uniform (min=0.5, max=0.9)
*f_I_*	Proportion of infected individuals in the initial condition	Uniform (min=0.005, max=0.02)
*f_R_*	Proportion of recovered individuals in the initial condition	1-*f_S_*-*f_I_*
*ε_I_*	Number of symptomatic individuals isolated per day	Uniform (min=500, max=5000)
*c*	Number of contacts	Uniform (min=5, max=10)
*eff*	Selection efficacy	Uniform (min=0.2, max=0.8)
*r_AS_*	Asymptomatic-to-symptomatic ratio	Uniform (min=1/5, max=5/1)

## References

[1] Seade (Fundação Sistema Estadual de Análise de Dados) (2020). População do Estado de São Paulo.

[2] Jesus JG, Sacchi C, Candido DDS, Claro IM, Sales FCS, Manuli ER (2020). Importation and early local transmission of COVID-19 in Brazil, 2020. Rev Inst Med Trop Sao Paulo.

[3] Seade (Fundação Sistema Estadual de Análise de Dados) (2020). SP contra o novo coronavírus: boletim completo.

[4] Horton J (2020). Coronavirus: What are the numbers out of Latin America?. BBC News.

[5] Wilder-Smith A, Freedman DO (2020). Isolation, quarantine, social distancing and community containment: pivotal role for old-style public health measures in the novel coronavirus (2019-nCoV) outbreak. J Travel Med.

[6] Salathé M, Althaus CL, Neher R, Stringhini S, Hodcroft E, Fellay J (2020). COVID-19 epidemic in Switzerland: on the importance of testing, contact tracing and isolation. Swiss Med Wkly.

[7] Amaku M, Covas DT, Bezerra Coutinho FA, Azevedo RS, Stuchiner C, Wilder-Smith A (2021). Modelling the test, trace and quarantine strategy to control the COVID-19 epidemic in the state of São Paulo, Brazil. Infect Dis Model.

[8] Massad E, Amaku M, Wilder-Smith A, Costa Dos Santos PC, Struchiner CJ, Coutinho FAB (2020). Two complementary model-based methods for calculating the risk of international spreading of a novel virus from the outbreak epicentre. The case of COVID-19. Epidemiol Infect.

[9] Elzhov TV, Mullen KM, Spiess AN, Bolker B (2020). minpack.lm: R interface to the Levenberg-Marquardt nonlinear least-squares algorithm found in MINPACK, plus support for bounds. R package version 1.2-1.

[10] Marino S, Hogue IB, Ray CJ, Kirschner DE (2008). A methodology for performing global uncertainty and sensitivity analysis in systems biology. J Theor Biol.

[11] Blower SM, Dowlatabadi H (1994). Sensitivity and uncertainty analysis of complex models of disease transmission an HIV model, as an example. Int Stat Rev.

[12] Kretzschmar ME, Rozhnova G, Bootsma MCJ, van Boven M, van de Wijgert JHHM, Bonten MJM (2020). Impact of delays on effectiveness of contact tracing strategies for COVID-19: a modelling study. Lancet Public Health.

[13] Bilinski A, Mostashari F, Salomon JA (2020). Modeling Contact Tracing Strategies for COVID-19 in the Context of Relaxed Physical Distancing Measures. JAMA Netw Open.

